# Components of Volatile Fractions from *Eucalyptus camaldulensis* Leaves from Iraqi–Kurdistan and Their Potent Spasmolytic Effects

**DOI:** 10.3390/molecules25040804

**Published:** 2020-02-13

**Authors:** Dlzar A. Kheder, Omar A. M. Al-Habib, Gianluca Gilardoni, Giovanni Vidari

**Affiliations:** 1 Biology Department, Faculty of Science, University of Zakho, Duhok 42001, Kurdistan Region, Iraq; habbibomar@yahoo.com; 2 Department of Chemistry, University of Pavia, Via Taramelli 10, 27100 Pavia, Italy; 3 Department of Biology, College of Science, International University of Erbil, Erbil 44001, Kurdistan Region, Iraq; 4 Departamento de Química y Ciencias Exactas, Universidad Técnica Particular de Loja, San Cayetano Alto s/n, Loja 1101608, Ecuador; 5 Medical Analysis Department, Faculty of Science, Tishk International University, Erbil 44001, Kurdistan Region, Iraq

**Keywords:** *Eucalyptus camaldulensis*, Myrtaceae, volatile fractions, Kurdistan traditional medicine, relaxant effects on rat contracted aorta and tracheal rings, calcium channels

## Abstract

Inhalation of vapors from a hot tea of *Eucalyptus camaldulensis* Dehnh. leaves is considered by Iraqi–Kurdistan people an effective spasmolytic and antipyretic remedy for the treatment of respiratory diseases. The constituents of volatile fractions isolated by hydrodistillation from dried leaves of the plant collected in Kurdistan were determined by GC-FID and GC-MS analyses. More than 90% components were identified. The most abundant constituents were 1,8-cineole, *p*-cymene, α-pinene, terpinen-4-ol, aromadendrene, and α-terpineol. The different volatile fractions induced relaxation on rat isolated aortic and tracheal rings in concentration-dependent manner. These effects appeared to be due to a complex interaction between various terpenoid components rather than being only due to the main oil constituent, 1,8-cineole. The KCa channel and the NO pathway were not significantly involved in the relaxation mechanism, while Ca^2+^ channels played a major role in the spasmolytic effects.

## 1. Introduction

The Kurdistan region of Iraq has a rich biodiversity and many medicinal herbs, including *Eucalyptus* species, are used by local people in traditional medicine since time immemorial [1]. A common antispasmodic and antipyretic remedy for treating respiratory tract diseases is prepared with the leaves of *Eucalyptus camaldulensis* Dehnh. (family Myrtaceae), also known as river red gum or Murrey red gum tree [2]. A hot tea is made and then the vapors from the aromatic infusion are inhaled by sick people. The empirical knowledge of this traditional medicine is passed on by oral tradition. No scientific rationale has been reported so far and the mechanism of action remains largely uninvestigated.

The leaf essential oils isolated from *E. camaldulensis* collected in such different countries as Egypt [3], Iran [4,5,6], Kenya [7], Montenegro [8,9], Pakistan [10,11], and Senegal [12], to cite only some of the most recent studies, have been investigated. Moreover, the leaf oil of *E. camaldulensis* has been reported to have potent antiseptic, antimicrobial properties [9,13,14], and it is commonly used in several countries for treating cough and cold, sore throat and other respiratory diseases [15]. Indeed, compounds with spasmolytic activity have been isolated from an EtOH extract of *E. camaldulensis* var. *obtusa* leaves [16]; however, the vasorelaxant effects of volatile fractions has been confirmed so far only by sporadic tests in vitro [17].

Moreover, previous studies point out that there is a considerable variation in the yields and chemical compositions of the volatile fractions isolated from different samples of *E. camaldulensis*.

For these reasons, it was worthy to study the components of volatile fractions isolated from *E. camaldulensis* leaves collected in Iraqi–Kurdistan using various isolation techniques. Furthermore, the physiological effects of volatile fractions on respiratory physiology were investigated in detail with the aim of sustaining the traditional use of this antispasmodic remedy with scientific evidence. To this purpose, the relaxant effects of different volatile fractions on the contractility of isolated rat aortic and tracheal rings have been determined. Moreover, in order to shed light on the mechanism of action, the involvement of the Ca^2+^ and K^+^ channels, and the nitric oxide (NO) pathway in the biochemical processes underpinning the spasmolytic activity of the plant, was tested in vitro.

## 2. Results and Discussion

Powdered air-dried leaves of *E. camaldulensis* were hydrodistilled in a standard Clevenger-type apparatus, by two different procedures, A and B, which are described in detail in Section 3.4. An oily volatile fraction, named EO_S_ was obtained by procedure A, named hydrodistillation with circulating-water. Three oils, named EO_W_, EO_A_, and EO_Ar_ were obtained by procedure B, named hydrodistillation with non-circulating water. Another crop of dried leaves was submitted to CO_2_ extraction at atmospheric pressure (see Section 3.5), which afforded an oily residue, named EO_D_. All the five oils had a pale yellowish color. They remained liquid even on storage at −20 °C in sealed vials and emanated a sharp and very distinct minty/pine smell. Each sample was dissolved in dichloromethane and analyzed by GC-FID and GC-MS.

### 2.1. Characteristics of the Volatile Fractions EO_S_ and EO_W_

The densities of EOs and EO_W_ at 22 °C were 0.95 and 0.93 g/mL, respectively. They were practically insoluble at 22 °C in H_2_O and highly soluble in EtOH, dichloromethane and dimethyl sulfoxide (DMSO). Therefore, oil stock solutions for biological tests were prepared in DMSO.

### 2.2. Chemical Analysis of Volatile Fractions (EO)

**Figure 1:**
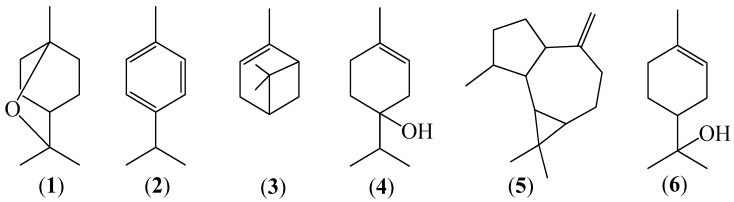
Structures of main components of leaf essential oil isolated from *E. camaldulensis* collected in the Iraqi–Kurdistan Region: 1,8-cineole (**1**), *p*-cymene (**2**), α-pinene (**3**), terpinen-4-ol (**4**), aromadendrene (**5**), and α-terpineol (**6**).

The chemical compositions of the five different oily volatile fractions (EO) obtained by hydrodistillation (procedures A and B) and by CO_2_ extraction, are reported in Table 1, together with the corresponding component percentages from GC-FID chromatograms and calculated linear retention indices (LRI). 31 components were identified in the volatile mixture EO_S_. Major components (see Figure 1) were 1,8-cineole (**1**, 62.70%), *p*-cymene (**2**, 6.70%), α-pinene (**3**, 4.71%), terpinen-4-ol (**4**, 3.86%), aromadendrene (**5**, 3.08%), and α-terpineol (**6**, 2.85%) which together accounted for the 83.9% EO_S_ (Table 1). Forty-two compounds were identified in EO_W_, among which 1,8-cineole (59.09%), *p*-cymene (6.55%), aromadendrene (3.97%), terpinen-4-ol (3.9%), α-pinene (3.75), and α-terpineol (2.94%) were the most abundant ones, accounting for the 80.2 % EO_W_. 35 compounds were identified in EO_A_ and the most abundant ones were 1,8-cineole (53.41%), terpinen-4-ol (6.96%), α-terpineol (6.70%), cryptone (6.69%), and spathulenol (2.57%). EO_Ar_, contained 40 identified components and it was enriched in α-terpineol (17.26%), cryptone (13.92%), *p*-cymen-8-ol (13.48%), 1,8-cineole (10.14%), and spathulenol (5.74%). EO_D_ contained only ten identified components, among which 1,8-cineole (89.25%), *p*-cymene (3.44%), and spathulenol (2.58%) were the most abundant ones.

Considering all the volatile fractions (Table 1), a total of sixty compounds were identified. They comprise 53 terpenoids, among which monoterpenoids (32) exceeded sesquiterpenoids (21) in number and total percentage. Oxygenated monoterpenoids (28) represented by far the most abundant family, followed by monoterpene hydrocarbons, whose number (4) was less than sesquiterpene hydrocarbons (12). 1,8-Cineole (eucalyptol) (**1**) was the most abundant oil component, followed by *p*-cymene (**2**), α-pinene (**3**), terpinen-4-ol (**4**), aromadendrene (**5**), and α-terpineol (**6**). Comparing the three different distillation procedures, hydrodistillation, either in mode A or B, afforded two oils, EO_S_ and EO_W_, in comparable yields, and having similar composition, and percentages of most components. In contrast, CO_2_ extraction was rather inefficient both in terms of oil (EO_D_) yield and chemical composition. In fact, only the rather non-polar compound 1,8-cineole (**1**) appeared to be removed from air-dried leaves in an efficient manner. The comparison of EO_A_ and EO_Ar_ with EO_W_ clearly showed that, as expected, the aqueous phase collected in the condenser was enriched in polar oxygenated monoterpenoids and sesquiterpenoids compared to non-polar terpenoid hydrocarbons. Specifically, borneol, terpinen-4-ol (**4**), cryptone, α-terpineol (**6**), and spathulenol exhibited a significant water solubility. Noticeable amounts of 1,8-cineole (**1**) were also dispersed in the aqueous phase. Moreover, comparison of EO_Ar_ with EO_A_ indicated that minor amounts of the most polar terpenoids were not completely removed from the aqueous phase by solid phase extraction (SPE, see Section 3.3).

Our data clearly showed that a significant quantity of oxygenated components remained dispersed in the aqueous phase collected in the condenser during leaf hydrodistillation. Therefore, we considered that a more accurate analysis of *E. camaldulensis* volatiles should include not only the components of the oil separated from the hydrosol, i.e., EO_W_, but also the mixture of polar compounds dispersed in the water condensed during the distillation process, i.e., EO_A_ and EO_Ar_. To this purpose, the column EO_Tot_ in Table 1 shows the sum of the percentages of each compound occurring in EO_W_, EO_A_, and EO_Ar_, averaged by the weight of each fraction. It thus resulted that the percentage of oxygenated monoterpenoids in the total volatile fraction EO_Tot_ was significantly higher than in the fraction EO_W_ alone, while that of hydrocarbons was lower. Only twelve components of EO_Tot_ had an abundance > 1%, namely, 1,8-cineole (**1**, 55.83%), *p*-cymene (**2**, 4.80%), terpinen-4-ol (**4**, 4.56%), α-terpineol (**6**, 4.38%), aromadendrene (**5**, 2.97%), cryptone (2.90%), α-pinene (**3**, 2.73%), spathulenol (2.08%), 9-*epi*-(*E*)-caryophyllene (1.91%), *p*-mentha-3,8-diene (1.66%), globulol (1.48%), and *p*-menth-1-en-7-al (1.02%), which together accounted for 86.32% EO_Tot_.

Phytochemical results indicate that three chemotypes of *E. camaldulensis* can be distinguished on the basis of the main components of the oil obtained by hydrodistillation under analogous conditions: one rich in 1,8-cineole (28–84%), one rich in *p*-cymene (20–30%) and one rich in spathulenol (18%) [8,22]. For example, the essential oil from *E. camaldulensis* leaves collected in Pakistan contained high concentration of oxygenated sesquiterpenes [11], while the main components of the leaf oil hydrodistilled from *E. camaldulensis* collected in Iran were *p*-cymene (68.43%), 1,8-cineole (13.92%), α-pinene (3.45%), and limonene (2.84%) [4]. Therefore, the compositions of EOs and EO_W_ clearly indicate that the variety of *E. camaldulensis* growing in Kurdistan can be classified as a chemotype with high 1,8-cineole and low *p*-cymene contents.

### 2.3. Relaxant Effects of E. camaldulensis Volatile Fractions on Aortic Rings

**Figure 2:**
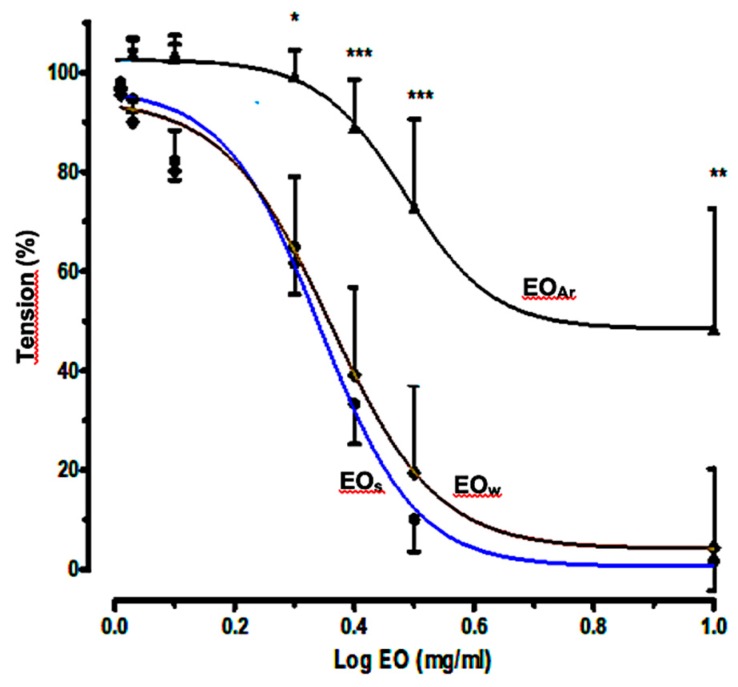
Concentration–response curves for the relaxant effects of volatile fractions EO_S_, EO_W_, and EO_Ar_ on rat aortic rings precontracted with phenylephrine (1 μM). * *p* < 0.05, ** *p* < 0.01 and *** *p* < 0.001.

In a preliminary study we showed that the essential oil of *E. camaldulensis* produced a potent dilation in aortic force development probably acting as a Ca^2+^ channel antagonism and partially via NO and cyclooxygenase pathways [17]. To investigate the relationship between oil composition and in vivo spasmolytic effects, the vasorelaxant effects of volatile fractions EO_S_, EO_W_, and EO_Ar_ on isolated rat aortic rings were determined separately, following the same procedure described previously [17]. The concentration–response curves for the oil-induced relaxation against phenylephrine (PE)-induced contractions are shown in Figure 2, while the percentages of relaxation, the values of Log EC_50_, and 95% CI (Confidence Interval) for Log EC_50_ are shown in Table 2. The curves show that the cumulative addition of the three samples at the plateau of contraction caused a relaxant effect in a concentration-dependent manner, with a strong potent dilation. Relaxation caused by EO_W_ did not differ significantly from EO_S_, as it could be anticipated by the comparable chemical compositions of the two oils; instead, significantly minor relaxation effects were observed for EO_Ar_. This finding seems to indicate that the reduced relaxant properties of EO_Ar_, compared to EO_S_ and EO_W_, is caused by the minor amount of 1,8-cineole (**1**) in EO_Ar_ (10.14%) than in EO_S_ (62.70%) and EO_W_ (59.09%). However, the physiological effects of EO_Ar_ were not totally suppressed, due to the significant presence of other active terpenoids (Table 1), such as α-terpineol (**6**, 17.26%) [23], spathulenol (5.74%) [24], carvacrol (2.73%), and thymol (2.52%) [25].

### 2.4. Relaxant Effects of EO_S_ and 1,8-Cineole (***1***) on Rat Tracheal Rings

**Figure 3:**
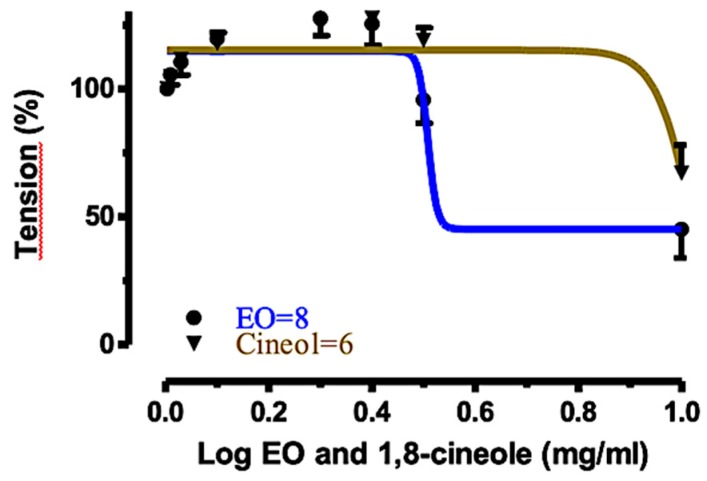
Concentration–response curves for the relaxant effects of 1,8-cineole (**1**) and EO_S_ on rat tracheal rings, precontracted with ACh (10 μM).

Since the oil EO_S_ showed the highest activity on rat aortic rings, compared to EO_W_ and EOAr, we deemed it to be interesting to measure the relaxant effect of this volatile fraction also in rat tracheal rings. Moreover, the major component of EO_S_, namely the monoterpenoid 1,8-cineole (**1**), was also tested in parallel experiments. In fact, it is well-known that 1,8-cineole (**1**) displays a wide range of biological effects, including muscle relaxant, bronchodilatatory [26,27,28,29], and anti-inflammatory [30,31] properties. Therefore 1,8-cineole (1) was used as the reference compound and its contribution to the biological activity of the volatile fraction EO_S_ was roughly estimated. The concentration–response curves for 1,8-cineole (**1**) and EO_S_ relaxant effects against acetyl choline (ACh)-induced contractions are shown in Figure 3. The percentages of relaxation were 33.01% and 79.44% for 1,8-cineole (**1**) and EO_S_, respectively. On the other hand, the Log EC_50_ were 1.030 and 0.508 (mg/mL), respectively. These results clearly indicated that both 1,8-cineole (**1**) and EO_S_ produced potent concentration-dependent relaxation in rat tracheal rings precontracted with ACh (10 μM), although the activity of EO_S_ was much higher than that of 1,8-cineole (**1**) alone. These findings nicely agree with the results of other authors who suggested that the relaxant [26,32] and cardiovascular [33] effects induced by *Eucalyptus* essential oils appeared to derive from a complex interaction between various terpenoid components rather than being due to a single compound.

### 2.5. Relaxation Mechanism

Aimed at clarifying possible molecular mechanisms involved in the relaxation induced by EO_S_ in tracheal rings, we explored the effects of EO_S_ on the roles of the K_Ca_ and Ca^2+^ channels, and on NO release. Actually, the crucial roles of K_Ca_ and l-type Ca^2+^ channels, as well as NO release in airway smooth muscle contraction are well known [17,23,25,26,27,28,29,33,34,35], and they are important therapeutic targets.

**Figure 4:**
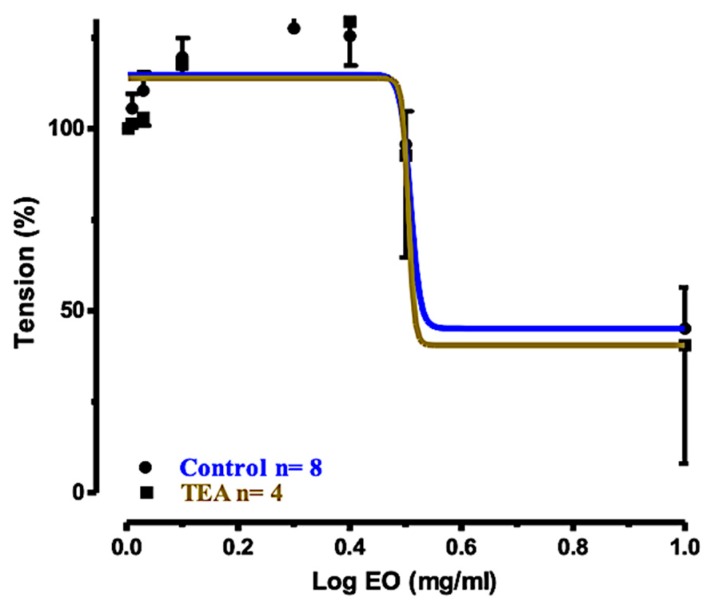
Concentration–response curves for the relaxant effects of EO_S_ on rat tracheal rings precontracted with ACh (10 μM): control and preincubated with TEA (1 mM).

The concentration–response curve for the effect exerted by EO_S_ against tracheal rings precontracted with ACh (10 μM) and preincubated with the calcium-activated K^+^ (K_Ca_) channel blocker tetraethyl ammonium chloride (TEA), is shown in Figure 4, in comparison with the curve of the control. The two curves were almost superimposed, indicating that the inhibition of the K_Ca_ channel was not a significant signaling pathway.

**Figure 5:**
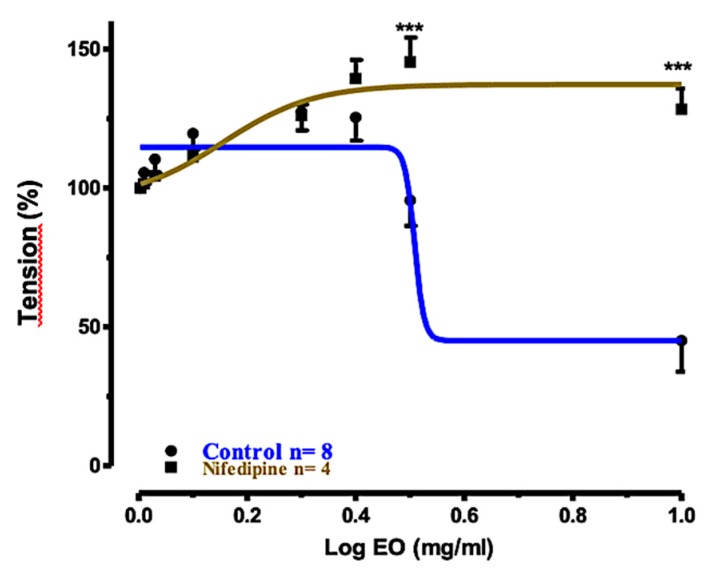
Concentration–response curves for the relaxant effects of EO_S_ on rat tracheal rings precontracted with ACh (10 μM): control and preincubated with nifedipine (30 μM). *** *p* < 0.001.

In another experiment, we measured the relaxation induced by EO_S_ in tracheal rings precontracted with ACh (10 μM) and preincubated with the L-type Ca^2+^ channel blocker nifedipine (30 μM). The corresponding concentration–response curve with respect to the control is shown in Figure 5. In tracheal smooth muscle, blockade of voltage dependent Ca^2+^ channels (VDCs) by nifedipine at the extracellular surface of the membrane may attenuate bronchoconstriction [34]. Spasm evoked by ACh and the maintenance of spontaneous tone largely depend on mechanisms for increasing the cytoplasmic concentration of free Ca^2+^ which are resistant to nifedipine [35]. In the event, the relaxation effect induced by EO_S_ was significantly reduced, suggesting that the presence of active constituents in the EO_S_ can cause tracheal relaxation via the inhibition of Ca^2+^ influx through the plasma membrane in tracheal smooth muscle.

**Figure 6:**
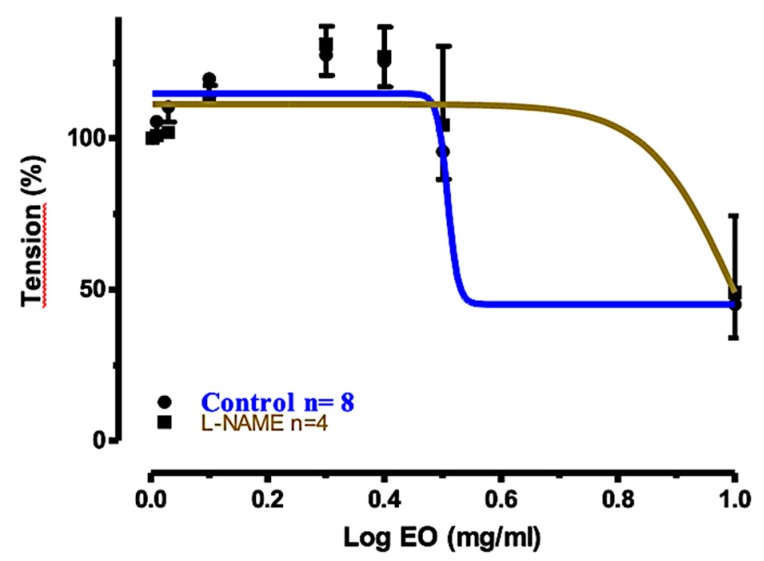
Concentration–response curves for the relaxant effects of EO_S_ on rat tracheal rings precontracted with ACh (10 μM): control and preincubated with L-NAME (0.3 mM).

In the last experiment, the relaxant effect of EO_S_ in tracheal rings precontracted with ACh and preincubated with NG-nitro-L-arginine methyl ester (L-NAME) was measured. L-NAME is a very well-known endothelial NO synthase inhibitor. The concentration–response curve of EO_S_, compared to the control, is shown in Figure 6. The graphic shows that the relaxation was attenuated to some extent; however, EO_S_ was still quite effective (45.41% relaxation).

Table 3 shows the log EC_50_, the 95% CI values, and the percentages of tracheal ring relaxation calculated from the concentration–response curves (Figure 4, Figure 5 and Figure 6) determined for EO_S_ in the three tests, compared to the control. In conclusion, these experiments clearly indicated that the pretreatment of tracheal rings with TEA or with L-NAME was unable to alter significantly the dose-dependent relaxant effects exerted by EO_S_. Therefore, the K_Ca_ channel and NO release and activation of the NO-cGMP pathway were not significantly involved in the relaxation mechanism. Instead, the inhibitory effect of EO_S_ in tracheal rings preincubated with nifedipine blocked the concentration–response relaxation, indicating that L-type Ca^2+^ channels were significantly (P < 0.001) involved in the relaxation mechanism.

## 3. Materials and Methods

### 3.1. Plant Material

Fresh leaves of *E. camaldulensis* Dehnh. were collected in January 2010 near the Duhok Damp, in the Iraqi Kurdistan region, and were identified by Prof. Dr. Saleem Shahbaz, of the Herbarium of the Department of Biology, Faculty of Science, University of Zakho. The plant name has been checked with http://www.theplantlist.org on 08.24.2019. The plant is neither endangered nor protected in Kurdistan, and no specific permission was required for the collection. A voucher has been deposited at the Herbarium with the accession number AC031. The leaves were rinsed with tap water, air-dried for about twenty days at 20–25 °C in the shade, and ground in an electrical mill (IKA-WERKE, Staufen im Breisgau, Germany) to a fine powder just prior to extraction.

### 3.2. Chemicals

Heparin and racemic ketamine were purchased from Hikma Pharmaceuticals (Amman, Jordan); xylazine, phenylephrine hydrochloride, TEA, L-NAME from Sigma-Aldrich (St. Louis, MO, USA); nifedipine from Medochemie (Limassol, Cyprus); ACh from Acros Organics–Fisher Scientific (Madison, WI, USA).

### 3.3. Isolation of Volatile Fractions EO

Five volatile fractions EO were obtained as dense oils by two different procedures: (i) Hydrodistillation provided fractions EO_S_, EO_W_, EO_A_, and EOAr (see Section 3.3); (ii) CO_2_ extraction at atmospheric pressure afforded fraction EO_D_ (see Section 3.4). All samples were dissolved in dichloromethane and analyzed by GC-FID and GC-MS.

### 3.4. Hydrodistillation

Powdered air-dried leaves of *E. camaldulensis* were hydrodistilled in a standard Clevenger-type apparatus, by two different procedures, A and B. In procedure A, named *hydrodistillation with circulating-water*, condensed water was circulating, while water was not circulating in procedure B. In mode A, leaves (3 lots of 250 g each) were hydrodistilled and volatiles were collected in a cylindrical glass condenser, where an oily layer (labeled EO_S_) accumulated at the top of an aqueous phase. The latter could return by gravity to the distillation pot connected to the tip of the collector tube through a plastic tube. Water condensation and dipping in the pot were synchronized, so as to leave about 10 mL of an aqueous layer in the condenser. After 2.5 h of distillation no more oil apparently accumulated at the top of the aqueous phase. EO_S_ was carefully separated from the aqueous layer by a micropipette, dried over anhydrous Na_2_SO_4_, filtered using Whatman filter paper (No.1), and stored at −20 °C in a sealed vial until analysis. The average yield over 3 distillations was 0.98 ± 0.02% (*w*/*w*). This oil was considered as the reference essential oil of *E. camaldulensis* leaves. In mode B. named *hydrodistillation with non-circulating-water*, the leaves were hydrodistilled (3 repetitions from lots of 250 g each) while the total condensed aqueous phase remained in the collector tube during the entire distillation time (2.5 h). After 1 h from the end of distillation, two phases clearly separated in the condenser. The accumulated oily layer, labeled EO_W_ [average yield 0.88 ± 0.02% (*w*/*w*)] was carefully removed from the top of the hydrosol and treated as described for EO_S_. The condensed aromatic aqueous layer (Aq, about 750 mL) from each hydrodistillation was subjected to multiple solid phase extraction (SPE). To this purpose, a home-made cartridge was prepared by packing 10 g of Lichroprep RP-18 powder inside a glass syringe-like tube; subsequently, the reversed phase was washed with MeOH (50 mL), followed by deionized water (50 mL), and then dried under vacuum. Each fraction Aq was divided in 80 mL portions, which were individually subjected to SPE. Each portion was added to the top of the column, which was then drained out under vacuum, collecting the liquid (F). The column was then washed with MeOH (50 mL), and the eluate (E) was collected. The experiment was then repeated with a new portion of Aq, following an identical procedure. Subsequently, all F and E fractions were separately collected together and carefully evaporated under reduced pressure (200 mmHg) to give two oily residues, EO_Ar_ (average yield 0.05 ± 0.015%) and EO_A_ (average yield 0.28 ± 0.01%), respectively, which were stored under N_2_ in sealed vials at −20 °C until analysis.

### 3.5. CO_2_ Extraction

A sample of powdered dried leaves (250 g) was submitted to CO_2_ extraction at atmospheric pressure for 2 h, in a home-made glass equipment, according to the method described by Honkanen and Karvonen [36]. An oily residue (EO_D_, yield = 0.004%) was obtained which was stored under N_2_ in a sealed vial at −20 °C until analysis. Given the low yield, CO_2_ extraction was not repeated.

### 3.6. GC-FID Analysis

GC-FID analyses of the different volatile fractions (EO) were performed on a Perkin Elmer Auto system gas chromatographer equipped with a HP5 capillary column (25 m × 0.32 mm i.d.; 0.52 µm film thickness). Nitrogen was used as the carrier gas at a flow rate of 1.5 mL/min. The injector operated in split mode (split ratio 25:1) and was heated at 220 °C. The GC oven temperature was hold at 60 °C for 1 min, then increased by a gradient of 3 °C/min until 100 °C, hold at 100 °C for 30 min, then increased to 250 °C by a gradient of 10 °C/min, finally hold at 250 °C for 5 min. Each analysis lasted 64.3 min. One microliter of each oil (5% in CH_2_Cl_2_) was injected in each analysis. Percentages (%) of compounds > 0.01 occurring in each EO (Table 1) were calculated from the corresponding GC peak areas with respect to the total peak area in the GC-FID chromatogram without applying any correction factor. Three GC-FID analyses were performed for EO_S_ and EO_W_, while EO_A_, EO_Ar_, and EO_D_ were analysed only once.

### 3.7. GC-MS Analysis

GC-MS analyses of the different volatile fractions (EO) were performed using an Agilent Bench Top GC-MS equipment, comprising a 6890N network gas-chromatographic system combined with a 5973 Network Mass Selective Detector (Agilent Technologies, Wilmington, DE, USA), and equipped with a DB-5 glass capillary column (30 m × 0.25 mm i.d.; 0.25 µm film thickness). Helium was used as the carrier gas at a flow rate of 1 mL/min. Temperature of the injector: 220 °C; oven temperature program: isotherm at 60 °C for 1 min, then increased (3 °C/min) to 270 °C, followed by an isotherm at 270 °C for 5 min. Sample split ratio: 1:20; 1 µL of each oil (5% in CH_2_Cl_2_) was injected in each analysis. Mass spectra were acquired at 70 eV within a mass range of 41–350 Daltons (Da) with a scan time of 0.73 scans s^−1^; ion source temperature: 230 °C. Compound identification was at first based on their linear retention indices (LRIs). They were calculated according to Van Den Dool and Kratz [20], with reference to a homologous series of *n*-alkanes C_6_-C_20_ (TPH-6RPM of CHEM SERVICE), which were injected immediately after each oil analysis under the same gas-chromatographic conditions. Calculated LRIs were compared with the retention indices of authentic samples or literature data [18,19]. Comparison of the retention indices was considered reasonable in a range of ±20 units. The identity of each EO constituents was confirmed by comparing the mass spectral fragmentation patterns with those reported in the literature [18,19,21] and, whenever possible, with the GC-MS data of pure standard compounds.

### 3.8. Physiological Activities of Volatile Fractions

Experiments on rats were performed according to the Iraqi and institutional rules considering animal experiments, and in accordance with the internationally accepted principles for laboratory animal use and care as found in the European Community guidelines (EEC Directive of 1986; 86/609/EEC). Stock solutions (0.05% g/L) of tested volatile fractions and 1,8-cineole were prepared by dissolving the desired amount in DMSO in which samples are completely soluble and which helped dissolution in water. They were kept refrigerated until analysis. Desired serial dilutions were then prepared by diluting stock solutions with aqueous NaCl (0.09% g/L) and warmed to 37 °C prior to use.

### 3.9. Measurement of Isometric Force with Isolated Thoracic Aortic Rings

Adult male albino rats, weighting 200–300 g each, were used for all experiments. They were kept in plastic cages at 24 °C and were exposed to a photoperiod cycle of 12 h light followed by 12 h darkness. Rats were fed standard diet and tap water. Access to water was free, while food was removed 24 h prior to experiments. Rats were injected intraperitoneally with heparin (1500 units/Kg body weight), then left for 30 min to avoid blood clotting and possible damage of the aorta endothelium, and finally anaesthetized with racemic ketamine (40 mg/kg) and xylazine (10 mg/kg) intraperitoneally. Chest cavity was opened, and excess tissues and fat were removed. Aorta was isolated and transferred to a beaker containing a Krebs solution aerated with 95% O_2_ and 5% CO_2_, that was placed in a water-bath at 37 °C. Subsequently, the aorta was segmented into rings 3–5 mm long. Isolated thoracic aortic rings were used in preparations with intact endothelium. Aortic rings were mounted between two stainless steel hooks, connected by a thread to a force transducer coupled to a transbridge amplifier and a Power Lab Data Acquisition system (model ML 870, Power Lab, AD Instrument, Sydney, Australia), which was connected to a computer running chart software (Version 7). The isometric force produced was monitored and recorded. Experiments were performed in 10 mL organ baths filled with a physiological Krebs solution (pH = 7.4) maintained at 37 °C by means of a thermoregulating system with water continuously circulating throughout a double-walled water-jacketed system, and continuously gassed with 95% O_2_ and 5% CO_2_. Tension was set at 2 g for 60 min, and the buffer solution was changed every 15 min until the resting tone became constant. After these numerous washings most xylazine was considered to have been removed from the organ so that its effect on the tissue was negligible. Sample concentration–response curves were then determined, with phenilephrine (1 μM) used as the aorta ring-contraction agonist.

### 3.10. Measurement of Isometric Force with Isolated Tracheal Rings

Trachea was removed from anaesthetized rats, cleaned, and segmented into 3–5 mm long rings (each containing 3–4 cartilaginous rings). Measurements of the isometric force with isolated tracheal rings were then performed according to international standard procedures for in vitro study using organ bath. Rings were suspended in 10 mL organ baths filled with physiological Krebs solution (pH = 7.4), maintained at 37 °C by a thermoregulating system with continuous water circulating throughout a double walled water jacket system, and aerated with 95% O_2_ and 5% CO_2_. Tracheal rings were maintained under an isometric tension of 2 g and allowed to stabilize for 60 min, while changing the Krebs solution every 15 min. After these numerous washings most xylazine was considered to have been removed from trachea so that its effect on the tissue was negligible. Concentration–response curves (Figure 3, Figure 4, Figure 5 and Figure 6 and Table 3) were then determined adding increasing amounts of EO_S_ and 1,8-cineole, in separate experiments. ACh (10 μM) was used as the tracheal ring-contraction agonist. In subsequent experiments, increasing amounts of EO_S_ were added to tracheal rings preincubated with the K^+^ channel blocker TEA (1 mM) for 20 min and then precontracted with ACh (10 μM). Analogous experiments were performed with tracheal rings preincubated with nifedipine (30 μM) for 10 min prior to precontraction with ACh (10 μM). Finally, tracheal rings were preincubated with L-NAME (0.3 mM) for 10 min. prior to precontraction with ACh (10 μM); then, increasing amounts of EO_S_ were added.

### 3.11. Statistical Analysis

The vasorelaxation response, calculated as a percentage of contraction produced by PE or ACh, was expressed as the mean ± standard error of the mean (SEM). The base line tension was expressed as a measure of 100% relaxation, and the tension induced by agonist was taken as a measure of 0% relaxation. All data analyses were fitted with a Hill equation, that the median effective concentration (Log of IC_50_) value was given as geometric mean with 95% confidence intervals (95% CI), using the statistics program GraphPad Prism^™^ software, version 6 (GraphPad Software, Inc., San Diego, CA, USA). Two-way analysis of variance (ANOVA) was performed, supported with Bonferroni test when carrying out pairwise comparison between the same doses of different groups using GraphPad program. *p*-Values less than 0.05 (*p* < 0.05) were considered significant. Symbols * mean *p* < 0.05, ** *p* < 0.01 and *** *p* < 0.001 for all graphs.

## 4. Conclusions

In this study the chemical profiles of the volatile fractions isolated from air-dried leaves of *E. camaldulensis* Dehnh. collected in Iraqi Kurdistan, were investigated for the first time. Essential oils EO_S_ and EO_W_, respectively, were obtained by hydrodistillation with circulating water and with non-circulating-water, respectively. They were compared with an oil (EO_D_) obtained by CO_2_ extraction. The first two methods were much more efficient in terms of oil yield and number of oil components. A total of 31 and 42 components have been identified and quantified in EO_S_ and EO_W_, respectively, by GC-FID and GC-MS, and comparison with literature databases. They accounted for more than 98% of the contents of the two oils. In both EO_S_ and EO_W_, monoterpenoids prevailed over sesquiterpenoids, and oxygenated monoterpenes were more abundant than monoterpene hydrocarbons. The major components of EO_S_ and EO_W_, accounting for more than 80%, were 1,8-cineole (**1**, about 60%), followed by *p*-cymene (**2**), α-pinene (**3**), terpinen-4-ol (**4**), aromadendrene (**5**), and α-terpineol (**6**).

In conclusion, the volatile fractions isolated from *E. camaldulensis* leaves collected in Kurdistan contained important and widely used flavor and fragrance ingredients. Most constituents, mainly 1,8-cineole (**1**) and other oxygenated monoterpenoids, are biologically active, exhibiting well-known antibacterial, bronchodilatory, anti-inflammatory, and analgesic effects [2,5,26,27,28,29,30,31,32,33,37,38]. In this regard, we have shown that the relaxant activity of the volatile fractions does not depend only on the most abundant constituent, 1,8-cineole (**1**), but it is likely due to the synergistic effects of different monoterpenoids. An important pathway for the relaxation effects exerted by *E. camaldulensis* volatile fractions involves the inhibition of Ca^2+^ influx through the plasma membrane in tracheal smooth muscle.

We are all well-aware that the chemical composition of vapors inhaled from a leaf hot tea can be different from that of a volatile fraction isolated by hydrodistillation, such as EO_S_, and should be analyzed by techniques such as solid phase microextraction (SPME)-GC-MS. However, it appears plausible that most of the bioactive components of the EOs also occur in the vapors, although the relative percentages can be different. Thus, based on our findings, we believe that inhalation of vapors from a hot aqueous infusion of *E. camaldulensis* leaves for treating the symptoms of respiratory tract diseases, is sustained by scientific evidence.

## Figures and Tables

**Table 1:** Chemical components and their percentages (%) in the different volatile fractions (EO) isolated from *E. camaldulensis* leaves ^a^.

Component	*RI* ^b^	*LRI* ^c^	EO_S_^d^	EO_W_^d^	EO_A_	EO_Ar_	EO_Tot_^e^	EO_D_
Isovaleric acid ^f^	848	842	‒	‒	‒	0.23	< 0.01	‒
Isopentyl acetate	869	870	0.07	0.07	‒	‒	0.05	‒
Undetermined		919	0.06	0.02	‒	‒	0.01	‒
α-Pinene ^g^ (**3**)	932	925	4.71	3.75	‒	‒	2.73	1.89
4-Methylvaleric acid ^f^	949	946	‒	‒	‒	0.01	<0.01	‒
Hexanoic acid ^f^	990	993	‒	‒	‒	0.01	<0.01	‒
*p*-Cymene (**2**)	1020	1024	6.70	6.55	0.16	‒	4.80	3.44
1,8-Cineole ^g^ (**1**)	1026	1031	62.70	59.09	53.41	10.14	55.83	89.25
Phenylacetaldehyde ^f,g^	1047	1048	‒	‒	0.11	0.06	0.03	‒
*p*-Mentha-3,8-diene	1068	1065	2.44	2.28	‒	‒	1.66	1.32
*p*-Cymenene	1089	1090	0.15	0.17	0.13	0.13	0.16	‒
Isopentyl isovalerate	1102	1107	0.28	0.38	‒	‒	0.28	0.15
*endo*-Fenchol	1114	1110	0.14	0.21	‒	‒	0.15	‒
*cis*-*p*-Menth-2-en-1-ol	1118	1120	‒	‒	0.16	0.22	0.05	‒
α-Campholenal	1122	1122	0.05	0.18	0.36	0.6	0.24	‒
*trans*-Pinocarveol	1135	1138	0.94	0.95	‒	3.55	0.83	‒
*trans-p*-Menth-2-en-1-ol	1136	1140	0.13	0.03	‒	0.16	0.03	‒
Sabina ketone	1154	1151	0.05	0.04	1.93	0.03	0.48	‒
Pinocarvone	1160	1162	0.62	‒	‒	‒	‒	‒
Umbellulone	1167	1162	‒	0.31	0.23	0.01	0.28	‒
Borneol^g^	1165	1166	0.09	0.47	1.47		0.68	‒
Terpinen-4-ol ^g^ (**4**)	1174	1177	3.86	3.90	6.96	2.70	4.56	0.39
Cryptone	1183	1184	1.34	1.10	6.69	13.92	2.90	‒
*p*-Cymen-8-ol	1179	1187	‒	‒	‒	13.48	0.53	‒
α-Terpineol ^g^ (**6**)	1186	1189	2.85	2.94	6.70	17.26	4.38	0.09
Undetermined		1213	0.25	0.25	‒	0.01	0.18	‒
*trans*-Carveol ^g^	1215	1219	0.14	0.15	0.65	1.02	0.30	‒
*cis*-Carveol ^g^	1229	1222	‒	‒	0.91	2.07	0.29	‒
*cis-p*-Mentha-1(7),8-dien-2-ol	1227	1227	‒	0.19	1.21	2.12	0.50	‒
(*E*)-Ocimenone	1235	1231	0.19		1.73	3.81	0.55	‒
Cuminaldehyde	1238	1238	0.11	0.12	0.32	0.39	0.18	‒
Carvone ^g^ + carvotanacetone	1239-1244	1244-1246	‒	‒	0.32	0.55	0.10	‒
Piperitone ^g^	1249 ^a^,1254 ^d^	1254	0.11	0.11	0.47	0.92	0.23	‒
*p*-Menth-1-en-7-al	1273	1274	0.73	0.68	1.67	3.41	1.02	‒
α-Terpinen-7-al	1283	1283	‒	‒	‒	0.01	tr	‒
Thymol ^g^	1289	1289	0.49	0.58	1.78	2.52	0.93	‒
*p*-Cymen-7-ol	1289	1292	‒	‒	0.24	0.49	0.07	‒
Carvacrol ^g^	1298	1302	0.39	0.44	1.15	2.73	0.69	‒
3-Oxo-*p*-menth-1-en-7-al	1330	1335	‒	‒	0.35	0.39	0.10	‒
Undetermined		1334	0.10	0.09	0.59	0.79	0.23	‒
Undetermined		1353	‒		0.17	0.11	0.04	‒
Isoledene	1374	1376	‒	0.09	‒	‒	0.06	‒
α-Copaene	1374	1378	‒	0.08	‒	‒	0.07	‒
β-Elemene	1389	1394	‒	0.03	‒	‒	0.02	‒
Undetermined		1419	‒	‒	0.13	0.06	0.03	‒
Undetermined		1434	‒	‒	0.17	0.05	0.04	‒
α-Gurjunene	1409	1412	‒	0.03	‒	‒	0.02	‒
β-Gurjunene	1433	1432	‒	0.04	‒	‒	0.03	‒
Aromadendrene (**5**)	1439	1443	3.08	3.97	0.3	0.15	2.97	0.47
9-*epi*-(*E*)-Caryophyllene	1464	1465	1.99	2.57	0.17	0.02	1.91	‒
γ-Gurjunene	1475	1476	‒	0.06	‒	‒	0.04	‒
γ-Muurolene	1478	1480	‒	0.43	‒	‒	0.31	‒
2-Phenylethyl 3-methylbutanoate	1490	1493	0.16	0.72	0.29	0.27	0.60	‒
Viridiflorene	1496	1499	‒	0.28	‒	‒	0.20	‒
Undetermined	1520	1534	‒	‒	0.10	0.22	0.03	‒
α-Muurolene	1500	1504	‒	0.28	‒	‒	0.20	0.42
γ-Cadinene	1513	1518	‒	0.07	‒	‒	0.05	‒
Palustrol	1567	1566	0.29	0.60	0.13	0.22	0.48	‒
Spathulenol	1577	1584	1.65	1.72	2.57	5.74	2.08	2.58
Globulol	1590	1590	1.65	1.96	0.18	0.29	1.48	‒
Viridiflorol	1592	1598	‒	0.48	‒	‒	0.35	‒
Isoaromadendrene epoxide ^f^	1612	1610	0.37	‒	0.20	0.23	0.05	‒
Undetermined		1630	0.21	0.45	0.13	0.19	0.36	‒
Occidenol	1676	1670	‒	‒	0.32	0.57	0.10	‒
Undetermined		1680	‒	‒	0.11	0.18	0.03	‒
Nootkatol	1714	1721	‒	‒	0.02	0.01	tr	‒
Undetermined		1727	‒	‒	0.74	1.64	0.24	‒
Undetermined		1751	‒	‒	0.68	1.53	0.22	‒
14-Oxy-α-muurolene	1767	1766	0.10	0.10	‒	‒	0.07	‒
Undetermined		1774	‒	‒	0.37	0.63	0.11	‒
Undetermined		1855	‒	‒	0.05	0.12	0.02	‒
11,12-Dihydroxyvalencene	1914	1908	‒	‒	0.20	0.55	0.07	‒
Monoterpene hydrocarbons (4) ^h^			14.00 (4)	12.75 (4)	0.29 (2)	0.13 (1)	9.35 (4)	6.65 (3)
Oxygenated monoterpenoids (28) ^h^			74.93 (18)	71.49 (18)	88.71 (22)	82.50 (25)	75.90 (27)	89.73 (3)
Sesquiterpene hydrocarbons (12) ^h^			5.07 (2)	7.93 (12)	0.47 (2)	0.17 (2)	5.88 (12)	0.89 (2)
Oxygenated sesquiterpenoids (9) ^h^			4.06 (4)	4.86 (5)	3.62 (7)	7.61 (7)	4.68 (9)	2.58 (1)
Others (7) ^h^			0.51 (3)	1.17 (3)	0.40 (2)	0.58 (5)	0.96 (7)	0.15 (1)
Undetermined (13) ^h^			0.62 (4)	0.81 (4)	3.24 (11)	5.53 (12)	1.54 (13)	0.00 (0)
Total % ^h^			98.57 (31)	98.20 (42)	93.49 (35)	90.99 (40)	96.77 (59)	100 (10)

^a^ Only peaks with a % > 0.01 have been considered; ^b^ *R*etention *I*ndex on a DB-5 column reported in the literature [18,19]; ^c^ *L*inear *R*etention *I*ndex on a DB-5 column calculated according to Van del Dool and Kratz [20]; ^d^ mean percentages of three analyses; ^e^ The EO_Tot_ value of each compound corresponds to the sum of the percentages in EO_W_, EO_A_, and EO_Ar_, respectively, averaged by the weight of each fraction; ^f^ tentatively identified by comparison with the *RI* and the mass spectra reported in databases [19,21]; ^g^ compound identity confirmed by coelution (GC) with an authentic sample; ^h^ Compound families and (total number) of compounds in each family over all EO’s.

**Table 2:** Relaxant effects of EO_S_, EO_W_, and EO_Ar_ on rat aortic rings precontracted with phenylephrine (1 μM).

	EO_S_	EO_W_	EO_Ar_
Log EC_50_	0.3384	0.3689	0.4852
95% CI for Log EC_50_	0.2974–0.3795	0.2627–0.4571	0.3621–0.6084
Relaxation (%) ± SEM	95.66 ± 0.198	88.17 ± 11.4	72.09 ± 10.61

**Table 3:** Relaxant effects of volatile fractions (EO_S_) in tracheal rings preincubated with K^+^ and Ca^2+^ channel blockers, and with NG-nitro-L-arginine methyl ester (L-NAME), respectively.

Treatment of Tracheal Rings	Control	Preincubated with TEA (1 mM)	Preincubated with Nifedizpine (30 μM)	Preincubated with L-NAME (0.3 mM)
Log EC_50_	0.508	0.505	0.156	1.014
95% CI for Log EC_50_	(Very wide)	(Very wide)	–0.0466 to 0.3577	(Very wide)
Relaxation (%) ± SEM	79.44% ± 9.101	73.68% ± 10.39	−26.42% ± 10.79	45.41% ± 10.31
